# Xanthogranulomatous inflammation involving the gallbladder, bile duct, and pancreas: a case report

**DOI:** 10.3389/fonc.2023.1191181

**Published:** 2023-05-16

**Authors:** Jinchang Zhang, Haomin Lin, Xujia Li, Piao Wang, Xiaoli Yang, Bo Li, Qinxi Guo, Song Su

**Affiliations:** ^1^ Department of General Surgery (Hepatobiliary Surgery), The Affiliated Hospital of Southwest Medical University, Luzhou, Sichuan, China; ^2^ Department of Pathology, The Affiliated Hospital of Southwest Medical University, Luzhou, Sichuan, China

**Keywords:** xanthogranulomatous cholangitis, xanthogranulomatous pancreatitis, xanthogranulomatous cholecystitis, XGI, XGP

## Abstract

Xanthogranulomatous inflammation (XGI) is a rare, benign condition that can affect several organs, including the gallbladder, kidney, skin, gastrointestinal tract, lymph nodes, and soft tissues. It is often misdiagnosed as a malignancy. In this report, we present the case of a 79-year-old male who presented with persistent jaundice for 11 months. Computed tomography and magnetic resonance imaging revealed pancreatic head enlargement, gallbladder thickening, and common bile duct thickening, leading to a preoperative diagnosis of malignant neoplasm of the pancreatic head. During surgery, dense adhesions were found around the portal vein, suggestive of mass invasion. To relieve obstruction, choledochojejunostomy was performed. Postoperative pathological examination revealed xanthogranulomatous cholecystitis (XGCc), xanthogranulomatous cholangitis (XGCg), and xanthogranulomatous pancreatitis (XGP). XGI affecting the bile ducts and pancreas is extremely rare, and there are no reported cases of simultaneous involvement of the gallbladder, bile duct, and pancreas by XGI. This study provides valuable insight into the differential diagnosis of XGI by presenting the imaging features of XGI patients.

## 1 Introduction

Xanthogranulomatous inflammation (XGI) is a rare but well-described disease characterized by the accumulation of foamy macrophages and inflammatory cells in different parts of the body, with infiltration and adhesion (1, 2). XGI can infiltrate any organ but most commonly appears in the gallbladder and kidneys (1), called xanthogranulomatous cholecystitis(XGCc) and xanthogranulomatous pyelonephritis. XGI rarely infiltrates the pancreas and bile ducts. Only 34 cases of xanthogranulomatous pancreatitis (XGP) (3) (**Table 1**) and 15 cases of xanthogranulomatous cholangitis (XGCg) (4) (**Table 2**) have been reported in the English language literature (supplementary). To our knowledge, we report the first case of XGI with the simultaneous involvement of the gallbladder, bile duct, and pancreas.

**Table 1:** Summary of Reported Cases of XGP.

No./Ref/Year	Age/Gender	Symptoms	CA19-9	Solid vs Cystic	Size(cm)	Location	Preop-diagnosis	Surgery	Associated Lesions
1.Ueno et al, 1993(1)	42/M	Abd pain	Normal	Cystic	NA	Body	Pancreatic cysts	DP	None
2.Iyer et al, 2004(2)	36/M	Abd pain	NA	Solid	NA	Tail	Malignancy	DP	Abscess
3.Iyer et al, 2004(2)	50/M	Jaundice,Pruritus	NA	Solid	NA	Head	Malignancy	PD	Abscess
4.Kamitani et al, 2005(3)	82/M	Abd pain	Normal	Cystic	3	Tail	Malignancy	DP	IPMN
5.Okabayashi et al, 2007(4)	60/M	Abd pain,fever	NA	Cystic	NA	Tail	Pseudocyst	DP	None
6.Okabayashi et al, 2007(4)	69/M	Abd pain,fever	NA	Cystic	NA	Tail	Pseudocyst	DP	None
7. Iso et al, 2008(5)	82/M	Weight loss	NA	Solid	1.1×0.8	Tail	IPMC	DP	IPMC
8.Shima et al, 2008 (6)	66/M	Abd pain	High	Solid	4	Body	Malignancy	DP	None
9.Ikeura T et al, 2009(7)	73/M	Asymptomatic	Normal	Cystic	3.2×2.7	Body	Pancreatic cystic tumor	PD	None
10.Tanioka et al, 2009(8)	57/M	Abd pain,fever	NA	Cystic	2.5	Head	Malignancy	PD	None
11.Kim YN et al, 2010(9)	72/F	Asymptomatic	Normal	Cystic	1.5×1	Body	IPMN	PD	IPMC
12. Uguz et al,2010(10)	30/M	Abd pain	Normal	NA	NA	Head	Chronic pancreatitis	DP	None
13. Uguz et al,2010(10)	34/F	Abd pain	Normal	NA	NA	Head	Chronic pancreatitis	DP	None
14.Kim HS, 2011(11)	70/F	Abd pain	High	Solid	2.2×2.0	Head	Malignancy	PD	None
15.Nishimura et al, 2011(12)	76/M	Erythroderma,weight loss	Normal	Cystic	5.2×2.6	Body	IPMC	DP	None
16.Sonpal et al, 2012(13)	55/M	Abd pain	NA	Cystic	6×6	Head	Pancreatic pseudocyst	PD	None
17.Hanna et al, 2016(14)	50/F	Abd pain	High	Cystic	14×14×14.5	Body/Tail	Malignancy	DP	MCN
18.Atreyapurapu et al, 2016(15)	60/M	Abd pain, weight loss	Normal	Solid	NA	Head	Malignancy	PD	None
19.Park et al, 2016(16)	63/M	Abd pain, dyspepsia	Normal	Cystic	NA	Body/Tail	XGP	None	None
20.Kim J et al, 2016(17)	61/F	Abd pain	High	Cystic	6.2x6.0	Head	Malignancy	PD	None
21.Becker Weidman et al, 2017(18)	56/M	NA	NA	Cystic	2.5×1.5	Tail	Malignancy	DP	None
22.Gaur et al, 2017(19)	58/M	Abd pain, weight loss	High	Cystic and Solid	4.2 x3.9	Head	Malignancy	PD	None
23.Kwon HJ et al, 2017 (20)	64/M	Asymptomatic	NA	Cystic	1.8	Body	Malignancy	DP	None
24.Navarro et al, 2017(21)	80/M	Abd pain, weight loss	Normal	Solid	NA	Head	XGP	None	None
25.Kwon JH et al, 2018(22)	21–67/5 M, 5F	Abd pain	Normal	Solid (2) Cystic (6) Mixed (2)	2 to 11	Head (2) Body (2) Tail (6)	Pancreatic cancer(4),Pancreatitis related(2),pseudocyst(2),SPN(1), IPMN (1)	NA	None
26.Imokawa et al, 2021 (23)	80/F	Jaundice	Normal	Solid	2.0	Tail	IPMC	PD	IPMC

DP, Distal pancreatectomy; PD, pancreatoduodenectomy; IPMN, intraductal papillary mucinous neoplasm; IPMC, intraductal papillary mucinous carcinoma; MCN, mucinous cystic neoplasm; SPN, solid pseudopapillary neoplasm; Abd, Abdominal; NA, not available.

**Table 2:** Summary of Reported Cases of XGCg.

No./Ref/Year	Age/Gender	Symptoms	CA19-9	Solid vs Cystic	Size(cm)	Location	Preop-diagnosis	Surgery	Associated Lesions
1. Goldar-Najafi et al,2003(24)	56/F	Abd pain	NA	Solid	3.3×2.7	HD,liver	Malignancy	Liver resection	NA
2. Goldar-Najafi et al,2003(24)	45/M	Jaundice	NA	None	None	CBD	Malignancy	PD	NA
3.Pantanowitz et al,2004(25)	75/F	Cholangitis	NA	Solid	4.5×2.7×2.2	HD,liver	Malignancy	Hepatic segmentectomy, CCY	NA
4.Kawate et al,2006(26)	34/F	Jaundice	Normal	None	None	CBD,HD	Malignancy	Hepatectomy, extrahepatic bile duct resection	None
5.Krishna et al,2008(27)	55/M	Jaundice, cholangitis	NA	None	None	CBD,GB	Malignancy	CCY, CBD excision, RNY hepaticojejunostomy	XGCc
6.Krishna et al,2008(27)	43/F	Jaundice, abd pain,cholangitis	NA	NA	NA	HD,GB,common hepatic artery	Malignancy	CCY, RNY hepaticojejunostomy	XGCc
7.Krishna et al,2008(27)	48/F	Jaundice, cholangitis	NA	NA	2	CBD,ampulla	Malignancy	PD	XGCc
8.Krishna et al,2008(27)	56/M	Jaundice, cholangitis	NA	None	None	HD,GB	Malignancy	Hepatectomy, RNY hepaticojejunostomy	XGCc
9.Alsheik et al,2010(28)	37/M	Jaundice	NA	None	None	CBD,GB	NA	CCY	XGCc
10.Jun et al,2013(29)	67/M	Jaundice, abd pain	High	Solid	2.1	CBD,pancreas	Malignancy	PD	XGP
11.Garg et al,2014(30)	32/F	Jaundice, fever	NA	Solid	4×3	CBD,GB, liver	Malignancy	CCY with CBD excision,RNY hepaticojejunostomy	XGCc
12.Bae et al,2015(31)	80/M	Asymp	Normal	Solid	2	HD,liver	Malignancy	Left lobectomy	NA
13.Zbaida et al,2018(32)	9/M	Jaundice	NA	NA	NA	HD,liver	Malignancy	Resection of hilar mass,CCY,hepaticoduodenostomy	None
14.Ikezawa et al,2020(33)	70/M	Jaundice	High	None	None	CBD,GB	XGCc, cholangitis	None	XGCc
15.Zhang et al .2022 (34)	72/M	Abd pain, jaundice	High	None	None	HD,liver	Malignancy	Hepatectomy	None

HD, Hepatic duct; GB, Gallbladder, CCY, Cholecystectomy; CBD, Common bile duct; Asymp, Asymptomatic; Abd, Abdominal; RNY, Roux-en-y; PD, pancreatoduodenectomy; NA, not available.

## 2 Case report

**Figure 1:**
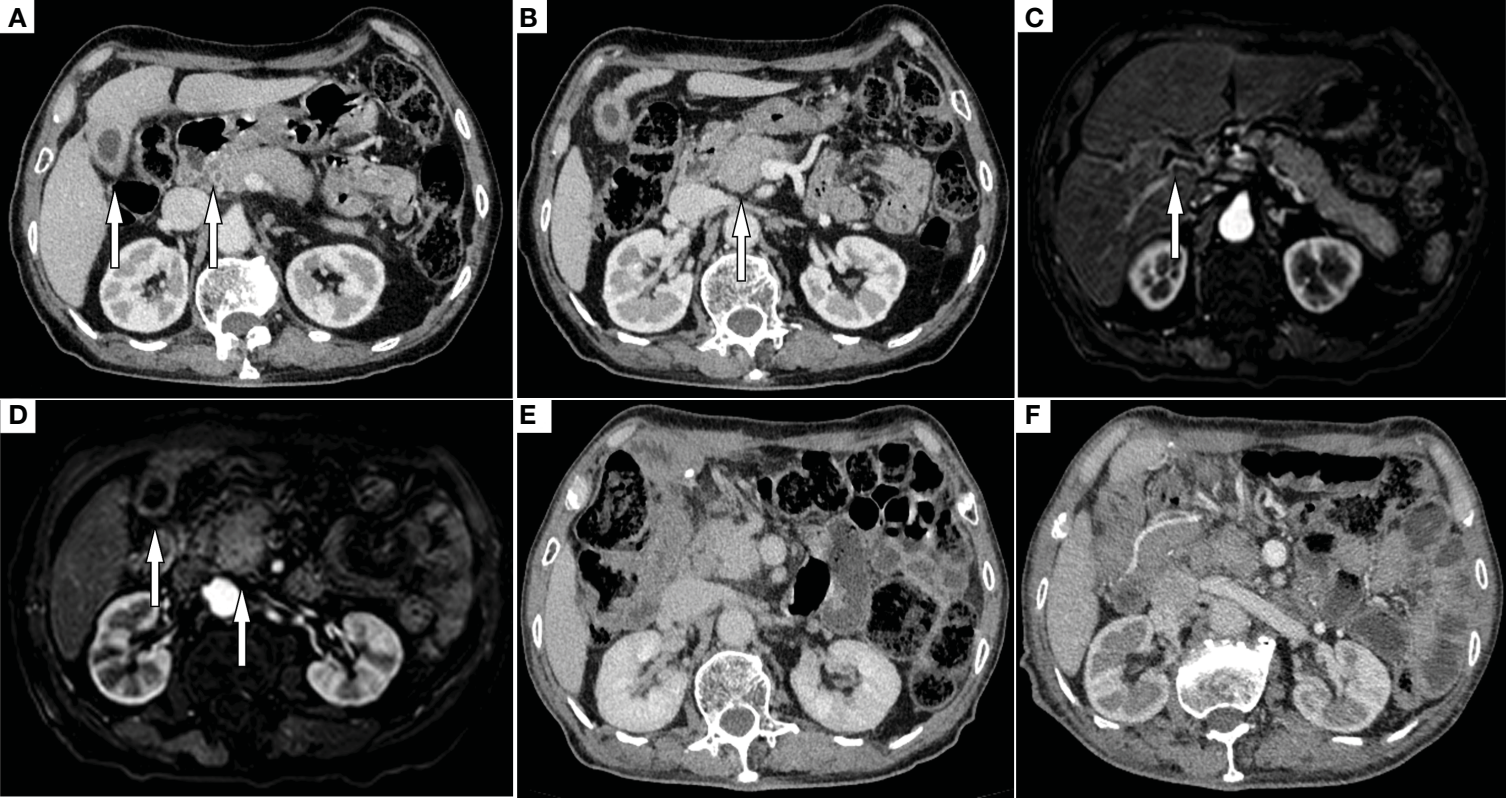
CT **(A)** Arrows indicate thickened gallbladder wall and thickened common bile duct; **(B)** Arrows indicate enlarged pancreatic head; MRI **(C)** Arrows indicate dilated extrahepatic bile ducts; **(D)** Arrows indicate thickened gallbladder wall and enlarged pancreatic head; **(E)** Follow up CT at 4 months after surgery; **(F)** Subsequent CT performed at 9 months post-surgery revealed no significant abnormalities in the pancreas.

A 79-year-old male was admitted to our hospital with abdominal pain, jaundice and weight loss. No obvious abnormality was found in the physical examination of the patient. Laboratory results: carbohydrate antigen 19-9 (CA19-9)>400 U/ml(normal range <37 U/ml), total bilirubin (TBIL) 267.1 μmol/L (normal range <23 μmol/L). Other laboratory test results were within the normal range. Because the patient had severe jaundice, he first underwent percutaneous transhepatic cholangio drainage. One month later, he was hospitalized again. Laboratory results: CA19-9 125.2 U/ml, TBIL 96.3 μmol/L. Contrast-enhanced computed tomography (CT) showed that the common bile duct was thickened (**Figure 1A**) and the head of the pancreas was enlarged (**Figure 1B**), but no abnormal enhancing shadow was found. Magnetic resonance imaging (MRI) showed dilatation of the extrahepatic bile ducts (**Figure 1C**) and thickening of the gallbladder wall. The pancreatic head was enlarged — the maximum transverse diameter was about 3.7 cm, the pancreatic signal was uniform, and no exact abnormal enhancement shadow was found (**Figure 1D**). On contrast-enhanced ultrasound, an uneven low-intensity lesion with a size of about 2.8 × 2.7 cm was found in the pancreatic head, with an irregular shape and an unclear boundary. We planned to make a further diagnosis with endoscopic ultrasound fine-needle aspiration, but the patient refused. Based on the above findings, the possibility of a pancreatic head malignant tumor was very high. Therefore, a pancreaticoduodenectomy was planned to be performed. During surgery, dilated and completely obstructed common bile ducts were found, and dense adhesions were present around the portal veins, which could not be divided. It was surmised that the mass invaded the portal vein. To relieve the obstruction, a choledochojejunostomy was performed, and some masses were removed for biopsy.

**Figure 2:**
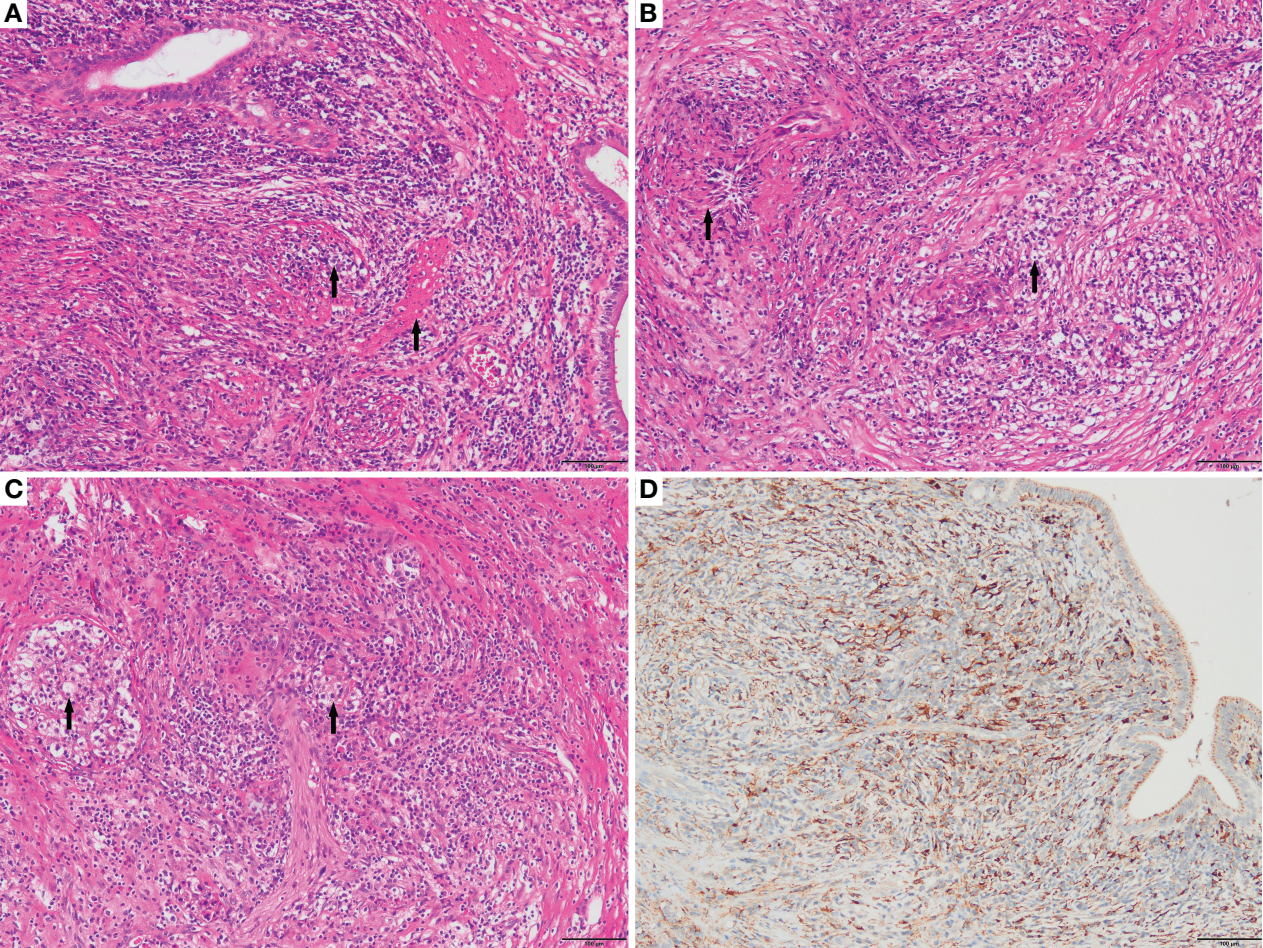
**(A)**. Interstitial fibroplasia of the gallbladder is accompanied by infiltration of foam cells, lymphocytes, and plasma cells. Arrows indicate the location of foam cells and the muscularis layer of the gallbladder from left to right (100×); **(B)**. Left-to-right pointing arrows denote the presence of pancreaticobiliary epithelial atrophy and foam cells, respectively (100×); **(C)**. Nodular aggregates of foam cells are visible, and all foam cells are indicated by the arrows(100×); **(D)**. Foam cell CD68+ (100×).

Numerous foam cell, lymphocyte, and plasma cell infiltrates were found in the resected mass and gallbladder (**Figures 2A-C**). Immunohistochemical findings showed CD68 (+) (**Figure 2D**), CK (-), VIM (+), CD19 (-), Ki-67 (<1%), ALK1 (-), and p53 (-). Immunohistochemical results supported the diagnosis of xanthogranuloma invading the pancreatic head, common bile duct, and gallbladder. After 10 months of close follow-up (**Figures 1E, F**), the patient had no symptoms and a good quality of life.

## 3 Discussion

XGI is a benign lesion, but it can be easily misdiagnosed as malignant and treated surgically. The pathogenesis underlying this condition remains unclear given the limited number of reported cases. The limited existing literature suggest that high intraluminal pressure and infection seem to be the etiology of the XGI series of disorders (3, 5). In the present case, it may be that XGCc occurred first because XGCc is more common and then inflammatory cells infiltrated into surrounding tissues along the biliary tract. This patient presented with progressive aggravation of jaundice for up to 11 months, which may account for such a wide range of infiltrates.

The diagnosis of most XGCg and XGP is achieved by the pathological evaluation of surgical or biopsy specimens. Therefore, we need to focus on the preoperative diagnosis of them. Although imaging findings and differential points of XGI have not been established, some features have been suggested to date. Zhao et al. (6) believe that diffuse gallbladder wall thickening, hypo-attenuated intramural nodules, continuous mucosal lines, lumen surface enhancement, and coexistence of gallstones highly suggest XGCc. In the present case, CT showed thickening of the gallbladder wall with continuous mucosal lines and the presence of an easily overlooked low-density image on the gallbladder wall. No gallstones were found, and parts of the CT features were the same as those reported by Zhao et al. (6). Krishna et al. (7) reported that a thick-walled gallbladder and bile duct stenosis might be indicators of XGCc with XGCg. In our CT features, a thick-walled gallbladder and thickened common bile duct were found. Therefore, the possibility of XGCc with XGCg should be considered when narrow or thickened bile ducts and thick-walled gallbladders occur together and simultaneously. XGP can develop from pancreatic pseudocyst or necrotizing pancreatitis. Early visible lobulated cystic masses can be misdiagnosed as solid pseudopapillary neoplasm, intraductal papillary mucinous neoplasm, and pseudocyst (8). Advanced imaging shows lobulated contours and heterogeneous masses, hypovascularity, and a progressive enhancement pattern with restricted diffusion, which are nearly identical to pancreatic cancer (9). Our case may be at an advanced stage.

Among treatment modalities, most previous cases were treated surgically, and only a few patients chose conservative treatment after diagnosis by one or more pathological examinations (10, 11). Currently, repeat pathological examination preoperatively is the only way to avoid surgical treatment. But repeat pathological examination preoperatively may also miss malignancy. Therefore, in the future, attention should be paid to the preoperative diagnostic methods for the XGI series of diseases.

## 4 Conclusion

In conclusion, although XGCg and XGP are rare diseases, they are often misdiagnosed as malignancies. Differential diagnosis should be emphasized. If CT simultaneously shows bile duct stenosis or thickening and gallbladder wall thickening, the possibility of XGI should be considered. Repeat pathological examination may be helpful in making the diagnosis.

## Data availability statement

The datasets presented in this study can be found in online repositories. The names of the repository/repositories and accession number(s) can be found in the article/supplementary material.

## Ethics statement

Written informed consent was obtained from the individual(s) for the publication of any potentially identifiable images or data included in this article.

## Author contributions

JZ and HL are responsible for the writing of this article, and XL collected patient data. QG, PW, XY, and BL for help with reviewing previous articles. SS was responsible for the final review of the article. All authors contributed to the article and approved the submitted version.
